# Fenretinide Acts as Potent Radiosensitizer for Treatment of Rhabdomyosarcoma Cells

**DOI:** 10.3389/fonc.2021.664462

**Published:** 2021-06-15

**Authors:** Eva Brack, Sabine Bender, Marco Wachtel, Martin Pruschy, Beat W. Schäfer

**Affiliations:** ^1^ Department of Oncology, Children’s Research Center, University Children’s Hospital Zurich, Zurich, Switzerland; ^2^ Children’s Research Center, University Children’s Hospital Zurich, Zurich, Switzerland; ^3^ Pediatric Hematology/Oncology, Department of Pediatrics, Inselspital, Bern University Hospital, University of Bern, Bern, Switzerland; ^4^ Department of Radiology Biology, University Hospital Zurich, Radio-Oncology, Zurich, Switzerland

**Keywords:** rhabdomyosarcoma, childhood cancer, fenretinide, radiation therapy, radiosensitizer, reactive oxygen species

## Abstract

Fusion-positive rhabdomyosarcoma (FP-RMS) is a highly aggressive childhood malignancy which is mainly treated by conventional chemotherapy, surgery and radiation therapy. Since radiotherapy is associated with a high burden of late side effects in pediatric patients, addition of radiosensitizers would be beneficial. Here, we thought to assess the role of fenretinide, a potential agent for FP-RMS treatment, as radiosensitizer. Survival of human FP-RMS cells was assessed after combination therapy with fenretinide and ionizing radiation (IR) by cell viability and clonogenicity assays. Indeed, this was found to significantly reduce cell viability compared to single treatments. Mechanistically, this was accompanied by enhanced production of reactive oxygen species, initiation of cell cycle arrest and induction of apoptosis. Interestingly, the combination treatment also triggered a new form of dynamin-dependent macropinocytosis, which was previously described in fenretinide-only treated cells. Our data suggest that fenretinide acts in combination with IR to induce cell death in FP-RMS cells and therefore might represent a novel radiosensitizer for the treatment of this disease.

## Introduction

Radiation therapy (RT) applying ionizing radiation (IR) is, along with chemotherapy and surgery, part of the standard therapeutic regimen for many malignancies. In the pediatric patient population this treatment is used e.g. in neuroblastoma, medulloblastoma, Ewing and soft tissue sarcomas (1). Rhabdomyosarcoma (RMS) is the most common soft tissue malignancy in children and young adolescents. Especially the fusion-positive rhabdomyosarcoma subgroup (FP-RMS) is associated with a poor outcome due to its aggressiveness and a high risk of relapse (2–5).

The effectiveness of IR is well studied and has direct and indirect effects on cancer cells. As direct action, IR damages DNA, proteins and lipids, which eventually results in genotoxic stress, cell cycle arrest and cell death (6). Indirect effects occur through radiolysis of water and the production of reactive oxygen species (ROS). The unpaired electrons in ROS are highly reactive and can induce DNA single- and double-strand breaks (7–10). Further, they act as signaling molecules driving cells towards cell death.

On the other hand, IR is also associated with considerable off-target effects and induces damage to non-diseased tissues and organs depending on the absorbed dose. This may cause relevant side effects especially in pediatric patients, which become apparent only later in life, such as growth retardation, reduced neurocognitive development, infertility, and most importantly the risk to develop secondary malignancies (11, 12). One current goal in radiobiology is therefore to minimize these side effects, while at the same time maximizing radiation benefits against tumor cells. Image-guided and intensity-modulated RT for example has led to significant improvements in the field (13, 14).

Another well-recognized option to achieve this goal is the simultaneous administration of radio sensitizers (15). Drugs are defined as radio sensitizing agents when they render cancer cells more vulnerable to radiation therapy. They have been categorized based on their structures into three different categories including small molecules, nanostructures, and macromolecules (16).

Previously, we identified the small molecule fenretinide (retinoic acid p-hydroxyanilide) as a potential additional treatment option for RMS, as it was found to have strong cytotoxic effects on FP-RMS cells (17). Fenretinide is a compound that is well established in the treatment for multiple malignancies during adulthood and that is already in clinical use in children (Clinicaltrials.gov ID NCT02163356) (18, 19). Importantly, its side-effect profile is very favorable with no limiting toxicities (20).

Multiple studies suggest that fenretinide induced cell death occurs mainly through apoptosis in most cell lines studied, either through the production of reactive oxygen species (ROS) or the involvement of lipid second messengers (21–25). In contrast, experiments in RMS showed that the underlying mechanism of cell death also depends on enhanced production of ROS and is accompanied by increased accumulation of cytoplasmic vesicles originating from macropinocytosis pathways (26), characteristics of a recently described new form of cell death (27, 28).

While fenretinide has not been investigated together with RT for the treatment of RMS, this combination is currently under investigation for the treatment of diffuse intrinsic pontine glioma (DIPG), with promising results in mice (29).

In the current study, we therefore elucidate the potential of fenretinide as radio sensitizer in RMS and describe the underlying mechanisms of cell death occurring during combination treatment in more detail. Overall, the study highlights the combination of fenretinide and IR as potential novel treatment option for FP-RMS.

## Material and Methods

### Gamma Irradiation

Irradiation was performed using an Xstrahl 200 kV X-Ray unit (Ratingen, Germany) at 100 cGy/min. Depending on the question, different intensities of radiation were applied to the cells.

### Cell Culture

The fusion-positive rhabdomyosarcoma cell line Rh4 (provided by Peter Houghton, Greehey Children’s Cancer Research Institute, San Antonio, Texas, USA) was maintained in high glucose Dulbecco’s Modified Eagle Medium (DMEM, Sigma-Aldrich, Buchs, Switzerland), supplemented with 100 U/ml penicillin/streptomycin (Invitrogen, ThermoFisher, Waltham, Massachusetts, USA), 2 mM L-glutamine (BioConcept, Allschwil, Switzerland) or Glutamax (Gibco, ThermoFisher, Waltham, Massachusetts, USA), and 10% fetal bovine serum (FBS, Sigma-Aldrich, Buchs, Switzerland), in 5% CO_2_ at 37°C. FP-RMS cell lines were regularly tested for mycoplasma infection, authenticated by short tandem repeat analysis (STR profiling) in 2011/2014 and positively matched with reference data (30).

### Cell Viability Assay

8,000 Rh4 cells were seeded in 96 well format (TC-Plate, Standard F, Sarstedt, Nümbrecht, Germany) in 100 µl medium. The studied compounds (see **Supplementary Table 7**) were added for 72 h. For measurement of cell viability, 10 µl WST-1 reagent (Sigma-Aldrich, Buchs, Switzerland) was added. After 30 min incubation at 37°C in the dark, absorbance at 440 and 640 nm were measured with a Synergy™ HT multi-detection microplate reader (BioTek, Winooski, Vermont, USA). The difference of the two values was calculated (delta optical density; ΔOD) and values from pure medium were subtracted as background.

### Clonogenic Assays

Clonogenic cell survival was determined by the ability of single cells to form colonies *in vitro* (31). 50,000 cells were seeded per 10 cm dish (TC-Dish, 100, Standard, Sarstedt, Nümbrecht, Germany). The following day, cells were treated with the desired concentration of fenretinide and irradiated with the desired intensity. After 12 days of culturing in 5% CO_2_ at 37°C the medium was removed and the cells fixed with glutaraldehyde (6.0%) and stained with crystal violet (CV) (0.5%). For data processing, the images were exported as TIFF files and the mean integrated density was quantified with the image processing program Fiji (53).

### Flow Cytometry

For all flow cytometry experiments, 150,000 Rh4 cells were seeded per well in Corning Costar 6-well plates (Sigma-Aldrich, Buchs, Switzerland). After treatments, cells were detached from the plates using trypsin, washed once with PBS and re-suspended in 0.5 ml indicated buffer. Data was acquired with the LSRII Fortessa flow cytometer (BD Biosciences, San Jose, California, USA) or the BD FACS Canto system (BD Biosciences, San Jose, California, USA).

Acquired data was analyzed with FlowJo software, version 9.9.6 (Tree Star Inc., Ashland, Orlando, USA). All used fluorescent stains are listed in **Supplementary Table 8** in **Supplementary Material and Methods.**

#### Pan ROS Measurement

Cells were seeded and treated with the desired compounds according to **Supplementary Table 7** (in the **Supplementary Material and Methods**). About 4 µM CellRox Deep red (ThermoFisher, Waltham, Massachusetts, USA) solution was simultaneously added to the medium. One hour after drug treatment, cells were irradiated with the desired intensity. After 18 h, cells were detached, washed in PBS and re-suspended in FluoroBrite live cell fluorescence imaging medium DMEM (ThermoFisher, Waltham, Massachusetts, USA). CellRox signal was measured by flow cytometry (50,000 events per sample) with excitation laser 640 nm and emission filter 670/14.

#### Mitochondrial ROS Measurement

Cells were seeded and treated with the desired compounds according to **Supplementary Table 7** in **Supplementary Material and Methods**.

One hour after drug treatment, cells were irradiated with the desired intensity. After 18 h, cells were detached, washed in PBS and re-suspended in MitoSox (ThermoFisher, Waltham, Massachusetts, USA) solution (10µM MitoSox in PBS) for 30 min at 37°C in the dark. MitoSox signal was measured by flow cytometry (50,000 events per sample) with excitation laser 561 nm, and emission filter 570 LP, 525/50.

#### Cell Cycle Analysis

Cells were seeded and treated with the indicated concentration of fenretinide. One hour after drug treatment, cells were irradiated with the desired intensity. After 24 and 48 h, cells were collected, washed with PBS and fixed with ice-cold 70% ethanol for 4 h at −20°C. Then cells were washed three times with PBS and incubated for 30 min with 20 mg/ml propidium iodide (PI) (Sigma-Aldrich, Buchs, Switzerland) and 200 mg/ml RNAse A (Qiagen, Hilden, Germany) in 0.1% Triton-X in PBS (Sigma-Aldrich, Buchs, Switzerland). PI signals were quantified by flow cytometry (50,000 events per sample) with excitation laser 488 nm, and emission filter 585/42.

#### Acridine Orange (AO) Staining

Acridine orange (Sigma-Aldrich, Buchs, Switzerland) (AO) was used to measure fluid-phase endocytic uptake induced by fenretinide treatment and IR after 48 h.

Cells were seeded and treated with the indicated compounds according to **Supplementary Table 7** in **Supplementary Material and Methods**. One hour after drug treatment, cells were irradiated with the desired intensity. After 48 h, AO (2.7 μM) in FluoroBrite DMEM live cell fluorescence imaging medium (ThermoFisher, Waltham, Massachusetts, USA) was added to the cells 4 h prior to their preparation for flow cytometry. Cells were then collected, washed in PBS and re-suspended in PBS. AO signal (50,000 events per sample) were acquired with excitation laser 488 nm and 561, emission filter 505 LP, 530/30 and 635LP, 670/30.

### Epifluorescence Microscopy

All images were taken with the Zeiss Axio Observer microscope (Zeiss, Oberkochen, Germany) equipped with a Hamamatsu Orca Flash 4.0 V2, sCMOS cooled fluorescence camera (Hamamatsu, Hamamatsu City, Japan) and an objective with 20× magnification. All fluorescent stains used can be found in **Supplementary Table 2** in **Supplementary Material and Methods**.

For data processing, images were exported as TIFF files and the mean integrated density was quantified with the image processing software Fiji (32). The integrated density value of an image was divided by the number of cells (counted on the phase image). Per treatment, a minimum of four pictures was taken.

#### Lucifer Yellow Fluorescence Microscopy

Some 50,000 cells per chamber were seeded in Falcon™ chambered cell culture slides (four wells, Corning) (Thermo Scientific, ThermoFisher, Waltham, Massachusetts, USA) and treated with 3 µM fenretinide. One hour after drug treatment, cells were irradiated with the desired intensity. After 48 h, cells were stained with Lucifer Yellow (820 µM) in FluoroBrite DMEM for 4 h at 37°C, 5% CO_2_. Afterwards, cells were washed with PBS and fixed with 4% PFA for 15 min at room temperature. After three PBS washes, the chamber was removed and the cells were mounted in Vectashield mounting medium with 4′,6-Diamidin-2-phenylindol (Vector Laboratories, Burlingame, California, USA).

### Western Blot

Whole cell extracts were prepared from cells lysed with RIPA buffer (50 mM Tris–Cl (pH 7.5), 150 mM NaCl, 1% NP-40, 0.5% Na-deoxycholate, 1 mM EGTA, 0.1% SDS, 50 mM NaF, 10 mM sodium β-glycerolphosphate, 5 mM sodium pyrophosphate, 1 mM sodium orthovanadate and supplemented with Complete Mini Protease Inhibitor cocktail (all from Sigma Aldrich, Buchs, Switzerland). Proteins were separated using NuPAGE™ Novex™ 4-12% Bis-Tris gels (ThermoFisher, Waltham, Massachusetts, USA) and transferred to nitrocellulose membranes (GE Healthcare Life Sciences). Membranes were blocked with 5% milk in TBS/0.05% Tween and subsequently incubated with primary antibodies overnight at 4°C. After three times washing in TBS-0.05% tween, membranes were incubated with horseradish peroxidase (HRP)-linked secondary antibodies for 1 h at RT.

Following antibodies were used: Rabbit anti-phospho-Histone H2A.X (Ser139) (Cat# 9718), rabbit anti-cleaved-Caspase 7 (Cat# 9491), rabbit anti-cleaved PARP (Cat# 5625), rabbit anti-GAPDH (Cat# 2118) all from Cell Signaling (Cell Signaling Technology, Danvers, Massachusetts, USA). Horseradish peroxidase-conjugated goat anti-rabbit antibody from Cell Signaling (Cat# 7077) were used as secondary antibodies. After three additional washing steps with TBS/0.05% Tween, proteins were detected by chemiluminescence using either the Pierce™ ECL Western blotting substrate (ThermoFisher, Waltham, Massachusetts, USA) or supersignal Western blotting reagent (ThermoFisher, Waltham, Massachusetts, USA) and a ChemiDoc MP (BioRad Laboratories AG, Cressier, Switzerland) imager. The images were analyzed with the software Image Lab Version 6.0. (BioRad Laboratories AG, Cressier, Switzerland).

### Statistics

The software GraphPad Prism (La Jolla, California, USA) was used for all statistical analyses. Comparisons of differences between two groups were analyzed by parametric paired t-test. The data were considered significant when p ≤0.05. Radiation/drug synergy was calculated using the Bliss independence model in the free Synergy Finder WebApp (33).

## Results

**Figure 1:**
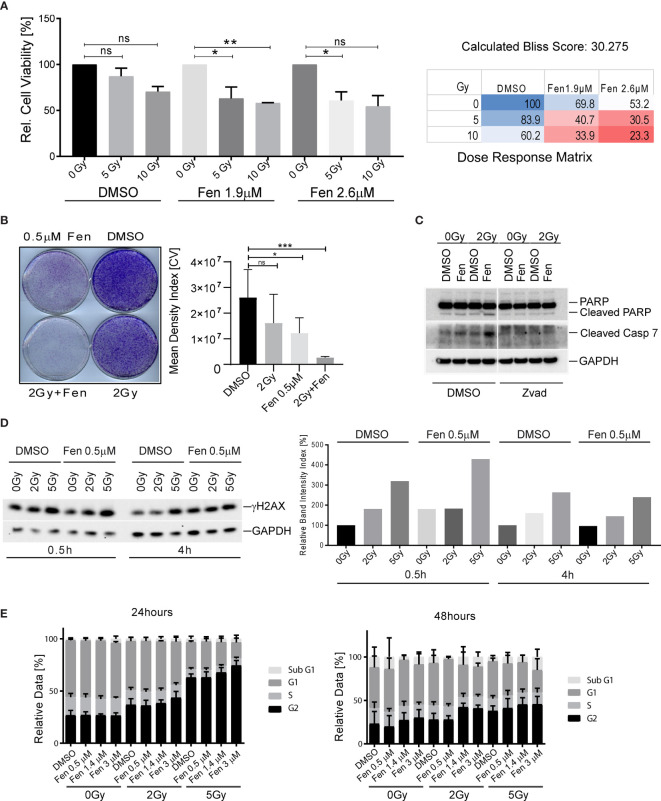
Combinatorial treatment of aRMS cells with fenretinide and ionizing radiation leads to enhanced cell death. **(A)** Cell viability of Rh4 cells treated with fenretinide in combination with ionizing radiation at the indicated concentrations and dosages as determined by WST assay (left panel). Synergy was calculated according to the Bliss independence model using the SynergyFinder WebApp (33) (right panel). ns: p > 0.05, *p</=0.05, **p</=0.01. **(B)** Clonogenicity assay with Rh4 cells treated with fenretinide and ionizing radiation at the indicated concentrations and dosages. Cells were grown for 12 days after treatment (left panel). Right panel shows the mean density index of the crystal violet (CV) stainings (n = 3). ns: p > 0.05, *p</=0.05, ***p</=0.001. **(C)** Western Blot using whole cell lysates from Rh4 cells treated with 0.5 μM fenretinide and 2 Gy IR. Cleaved PARP, Caspase 7 and GAPDH were detected. **(D)** Western Blot using whole cell lysates from Rh4 cells treated with 0.5 μM fenretinide and either 2 or 5 Gy IR. Phosph-H2AX and GAPDH were detected (left panel). Quantification of individual band intensities assessed by BioRad Software: Depicted are the normalized ratios of γH2AX and GAPDH (right panel). **(E)** Cell cycle analysis determined by flow cytometry of Rh4 cells after 24 and 48 h treatment with fenretinide and IR at the indicated concentrations and dosages. Staining with propidium iodide (20 mg/ml).

We previously demonstrated that fenretinide efficiently induces a novel dynamin-dependent cell death in FP-RMS cells (26). As RT is part of the FP-RMS standard treatment regimens, we questioned whether fenretinide would enhance the anti-tumor effect of RT on FP-RMS cells. In a first step, we therefore co-treated Rh4 cells with two single doses of radiation (5 and 10 Gy) and two low concentrations of fenretinide (IC10 and IC20, equals to 1.9 and 2.6 µM) and assessed cell viability after 72 h by WST-1 assay. Indeed, for all combination treatments, cell viability decreased, however only significantly for the lower concentration (**Figure 1A**, left panel, **Supplementary Table 1**). We found a dose dependent synergistic effect of fenretinide with IR, with calculated Bliss Scores of 30.275 according to the Bliss independence model [SynergyFinder WebApp (33)], indicating a very high synergistic effect (**Figure 1A**, right panel). Next, we assessed the combinatorial effect on clonogenic cell survival. In this setting, we observed a strong combinatorial effect already at lower concentrations of fenretinide (0.5 µM) together with low radiation doses of 2 Gy (**Figure 1B**, right and left panels).

Next, we investigated the mechanism of cell death that was induced by the combination treatment. Western blot analysis of cleaved Caspase 7 and cleaved PARP revealed induction of apoptosis in single treated cells, which was enhanced by the combination treatment. The addition of Z-vad, a pan-caspase inhibitor abolished both caspase 7 and PARP cleavage (**Figure 1C**). Next, we were interested to see whether the combination would induce enhanced phosphorylation of histone H2AX (γH2AX), a well-established marker for DNA double-strand breaks (34). Western blot analysis and the corresponding band intensity index showed enhanced phosphorylation of γH2AX in the combination treated cells, most prominently after 30 min and rapidly decreasing over the next 4 h (**Figures 1D**, left and right panel). Based on these findings, we further investigated the effect of fenretinide and IR on cell cycle distribution. Fenretinide alone did not change the cell cycle distribution after 24 h. In contrast, single treatment of IR induced a dose-dependent G2/M arrest (**Figure 1E**, left panel) which was further increased in the combination. After 48 h, the G2/M arrest was less prominent, but a Sub-G1 peak became evident indicating induction of cell death after this treatment period (**Figure 1E**, right panel). These data suggest that fenretinide combined with IR enhances a G2/M cell cycle arrest.

**Figure 2:**
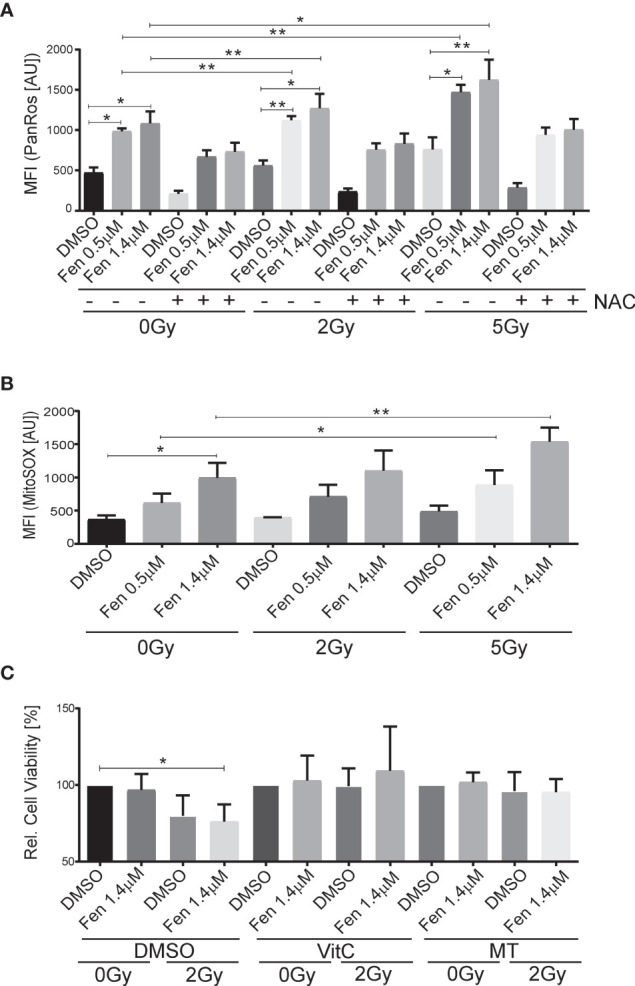
The combination of fenretinide and ionizing radiation enhances the production of reactive oxygen species. **(A, B)** Mean fluorescence index analysis of flow cytometry data of Rh4 cells treated with fenretinide (0.5/1.4 µM) and IR (2/5 Gy), in presence or absence of 15 mM N-acetylcysteine (NAC). Cells were stained with CellRox (4 µM) **(A)** and MitoSox (10 µM) **(B)**. *p</=0.05, **p</=0.01. **(C)** Cell viability of Rh4 cells treated with fenretinide (1.4 µM) and IR (2 Gy) in presence or absence of the mitochondria specific ROS scavenger MitoTempo (300 µM) or Vitamin C (50 µM) as determined by WST assay. *p</=0.05.

Next, we wanted to see whether the combination treatment affected generation of reactive oxygen species (ROS). Indeed, increasing concentrations of fenretinide and to a lower extent also increasing doses of IR enhanced ROS production (pan-ROS) compared to control cells. This was significantly more pronounced in the combination treatment (**Figure 2A** and **Supplementary Table 2**). To confirm specificity, we co-treated cells with the hydrogen-peroxide scavenger N-acetylcysteine (NAC) and observed a significant reduction of ROS production (**Figure 2A** and **Supplementary Table 2**). Since we previously found mitochondria derived ROS to be the main source of ROS under fenretinide treatment, we also analyzed cells with a mitochondria specific ROS staining (MitoSox). We observed a significant dose-dependent increase of mitochondrial ROS upon fenretinide treatment alone, as well as a further increase under combination treatment. Interestingly, radiation alone did not enhance mitochondrial ROS production (**Figure 2B** and **Supplementary Table 3**).

To further characterize and validate the impact of ROS species on cell death, we treated the cells additionally with Vitamin C as a well-recognized pan-ROS scavenger and MitoTempo, a mitochondrial-specific ROS scavenger. We observed an almost complete rescue from cell death by both Vitamin C and MitoTempo (**Figure 2C** and **Supplementary Table 4**).

Taken together, both fenretinide and IR induce the production of ROS whereas mitochondrial derived superoxides are mainly generated by fenretinide.

**Figure 3:**
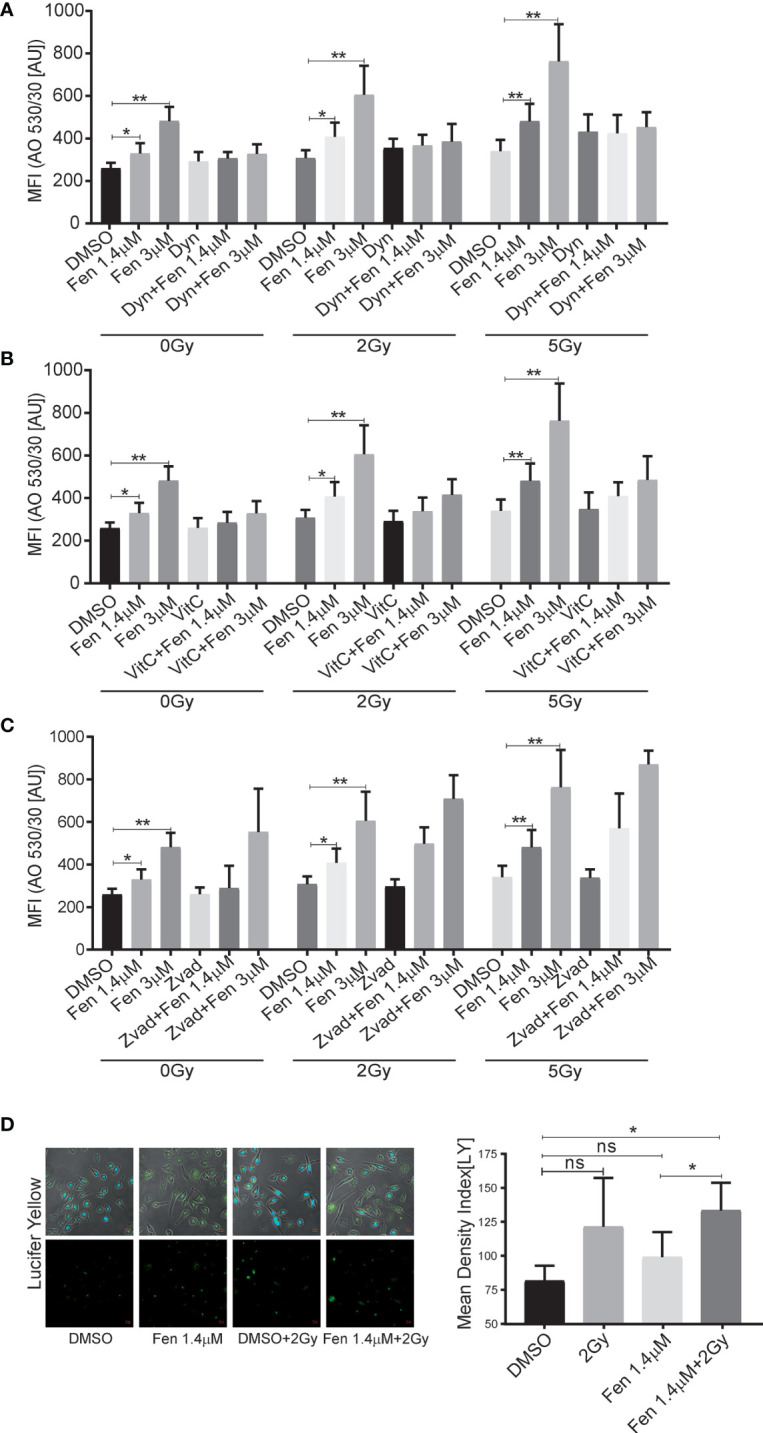
The combinatorial treatment of fenretinide and radiation therapy leads to an enhanced uptake of phase lucent dyes. **(A–C)** Mean fluorescence index analysis of flow cytometry data of fenretinide (1.4/3 µM) and IR (2 Gy/5 Gy) treated Rh4 cells, co-treated with either dynasore (30 µM) **(A)**, Vitamin C (50 µM) **(B)** or Z-vad (100 µM) **(C)** and stained with acridine orange (2.7 µM) using two different bandpass filters, here 530/30 (Bandpass filter 670/30 **Supplementary Figures 1A–C**) *p</=0.05, **p</=0.01. **(D)** Fluorescence microscopy images of Rh4 cells left untreated or treated with fenretinide (1.4 µM), IR (5 Gy) or the combination thereof and stained with Lucifer Yellow (820 µM). Quantification of the relative mean density index was performed with Fiji software: Total integrated density value of an image was divided by the number of cells. ns: p>0.05, **p</=0.01. **Supplementary Figure 1**: The combinatorial treatment of fenretinide and IR therapy leads to an enhanced uptake of phase lucent dyes. **(A–C)** Mean fluorescence index analysis of flow cytometry data of fenretinide (1.4/3 µM) and IR (2Gy/5Gy) treated Rh4 cells in presence or absence of dynasore (30 µM) **(A)**, Vitamin C (50 µM) **(B)** or Z-vad (100 µM) **(C)** and stained with acridine orange (2.7 µM) using two different bandpass filters, here 670/30 (Bandpass filter 530/30 **A–C**). **p</=0.01.

Previously, we could demonstrate that fenretinide induced the formation of large phase lucent cytoplasmic vesicles, which derive from increased macropinocytosis, an effect that could be efficiently blocked by the dynamin-inhibitor dynasore. Therefore, our next aim was to clarify whether fenretinide would also enhance accumulation of cytoplasmic vesicles when combined with IR. Hence, we co-treated cells with either dynasore, Vitamin C or Z-vad and measured acridine orange (AO) staining to assess endocytosis (**Figures 3A–C** and **Supplementary Figures 1A–C**). These experiments revealed a significant increase in dye uptake in the fenretinide-only treated cells, which was further enhanced by IR treatment. Interestingly, IR treatment alone only minimally affected the uptake of AO. In addition, co-treatment of the cells with Vitamin C and dynasore decreased the dye uptake in treated cells (**Figures 3A, B** and **Supplementary Figures 1 A, B** and **Supplementary Tables 5**, **6**). No change was observed in the IR-only treated cells (**Figures 3A, B**, **Supplementary Figures 1 A, B** and **Supplementary Tables 5**, **6**). As expected, the addition of Z-vad did not change the levels of endocytosis as measured by AO uptake (**Figure 3C**, **Supplementary Figure 1A** and **Supplementary Tables 5**, **6**).

Finally, to validate these findings we used the fluid phase dye Lucifer Yellow and performed fluorescence microscopy. We confirmed a non-significant increase of dye uptake when cells were treated with IR alone, whereas a significant increase was observed in the combination treatments (**Figure 3D**). These findings suggest that the combination of IR with fenretinide significantly enhanced the uptake of fluid phase dyes whereas IR alone does not. Further, enhanced endocytosis might depend on mitochondrial ROS production and involve dynamin GTPases as most likely triggering factors.

## Discussion

The aim of this study was to evaluate the potential of fenretinide as radio sensitizer for co-treatment of FP-RMS cells together with IR. In radiation therapy, timing, duration, and dose are crucial factors for effectiveness and prevention of long-term side effects. Therefore, identification of combinations of agents and treatment modalities that act synergistically is highly appreciated. Radiosensitizing agents are capable to broaden the therapeutic window and selectively augment radiation effects in tumor cells while simultaneously sparing the surrounding tissue. Fenretinide combined with IR was studied in the context of diffuse intrinsic pontine glioma (DIPG) and showed promising results in mouse experiments (29). However, up-to-now no other studies exploring this combinatorial effect in additional tumors have been performed.

We already showed that fenretinide has a strong anti-tumour effect in FP-RMS cells through the production of mainly mitochondria derived ROS, which induced a new form of a dynamin-dependent cell death accompanied by accumulation of cytoplasmic vesicles (26). Here, first experiments demonstrated a dose-dependent combinatorial anti-tumor effect of fenretinide together with IR. This enabled us to reduce both treatment dosages with a persisting effect already at 2Gy, which also impaired clonogenic growth.

As underlying cell death mechanism apoptosis was identified in part. However, treatment also led to induction of ROS and subsequent DNA damage. RT is known to induce G2 cell cycle arrest following DNA damage (35), which was confirmed by our findings. It is also known that fenretinide can induce cell cycle arrest (36). Our results showed that impaired cell cycle progression through G2/M is most pronounced upon combination treatment. This is an important finding as one of the hallmarks of cancer is sustained proliferative signaling, even after DNA damage (37), and therefore restoration of a normal physiological response such as induction of cell death is desirable.

Our experiments using a pan-ROS detecting agent further revealed a significant increase of ROS production in irradiated cells. In our previous experiments, we were able to show that fenretinide alone induces mitochondrial derived ROS (26). Here, irradiation mainly induced the production of hydrogen peroxide, which we were able to scavenge with NAC. Hence triggering different ROS species in our combination treatment might be important in the context of resistance development, as cancer cells are known to upregulate antioxidant pathways (38).

To identify the cell death mechanism in more detail, we evaluated whether IR would also trigger dynamin-dependent macropinocytosis as this was found previously to be a relevant mode of action of fenretinide in FP-RMS cells. As shown above, we were able to identify increased macropinocytosis in the co-treated cells. In cells irradiated only, this increase was minimal when assessed by flow cytometry but slightly more prominent when assessed by light microscopy. The discrepancy between flow cytometry and light microscopy might actually be an analysis bias and explained by the fact that ionizing irradiation induces cell cycle arrest and senescence (as observed by microscopy imaging) and subsequently morphological changes of cells. As they become bigger, they might be capable to take up more dye and the analysis will show an increased integrated mean density index per cell. Due to the gating strategy applied in flow cytometry, the cell size is not relevant. However, in contrast to cells treated with fenretinide alone, in cells treated only by IR dye uptake could neither be inhibited with a dynamin inhibitor nor with a ROS-scavenger. Based on these findings it is unlikely that IR induced cell death is the result of increased macropinocytosis. However, this cell death mode can be triggered and enhanced upon co-treatment with fenretinide, most likely through the induction of a distinct population of ROS.

Taken together our findings support the hypothesis that fenretinide acts as a promising radiation sensitizer in co-treatment of FP-RMS cells. Different modes of cell death mechanisms are activated and enhanced by these two treatment modalities. Reactive oxygen species and DNA damage are the main underlying triggering factors, whereas macropinocytosis as induced by fenretinide treatment plays only a minor role in IR-only treated cells. A combinatorial treatment with both modalities however may help to reduce development of resistances and increases the therapeutic window for local treatment. Hence, it might represent a promising treatment regimen in paediatric patients with FP-RMS.

## Data Availability Statement

The original contributions presented in the study are included in the article/**Supplementary Material**. Further inquiries can be directed to the corresponding author.

## Author Contributions

EB performed the experiments and wrote the manuscript. SB assisted her with the experiments. MW, MP, and BS provided the topic, supervised and guided the work, and proofread and edited the manuscript. All authors contributed to the article and approved the submitted version.

## Funding

This work was funded by Krebsliga Zürich.

## Conflict of Interest

The authors declare that the research was conducted in the absence of any commercial or financial relationships that could be construed as a potential conflict of interest.
