# Supplementary material for: Fenretinide Acts as Potent Radiosensitizer for Treatment of Rhabdomyosarcoma Cells
Source: Front Oncol. 2021 Jun 15;11:664462. doi: 10.3389/fonc.2021.664462 (PMC8239363; doi:10.3389/fonc.2021.664462)
Supplement: Supplementary file 1 [file DataSheet_1.docx]

**Supplementary Figure 1: The combinatorial treatment of fenretinide and IR therapy leads to an enhanced uptake of phase lucent dyes. A-C** Mean fluorescence index analysis of flow cytometry data of fenretinide (1.4/3 μM) and IR (2Gy/5Gy) treated Rh4 cells in presence or absence of dynasore (30 μM) **(A),** Vitamin C (50 μM) **(B)** or Z-vad (100 μM) **(C)** and stained with acridine orange (2.7 μM) using two different bandpass filters, here 670/30 (Bandpass filter 530/30 Figure 3 A-C), **: p</=0.01, ***: p</=0.001.

**Supplementary Tables**

**Statistical Analysis**

**Table 1** Statistical analysis of cell viability data using paired Students t-test

| **Treatment A** | **Treatment B** | **p-Value** | **Significance** |
| --- | --- | --- | --- |
| DMSO, 0Gy | DMSO, 5Gy | 0.1313 | ns |
| DMSO, 0Gy | DMSO, 10Gy | 0.0826 | ns |
| DMSO, 5Gy | Fen 1.9 µM, 5Gy | 0.0150 | * |
| DMSO, 5Gy | Fen 2.6 µM, 5Gy | 0.0001 | *** |
| DMSO, 10Gy | Fen 1.9 µM, 10Gy | 0.1813 | ns |
| DMSO, 10Gy | Fen 2.6 µM, 10Gy | 0.4102 | ns |
| Fen 1.9 µM, 0Gy | Fen 1.9 µM, 5Gy | 0.0365 | * |
| Fen 1.9 µM, 0Gy | Fen 1.9 µM, 10Gy | 0.0033 | ** |
| Fen 2.6 µM, 0Gy | Fen 2.6 µM, 5Gy | 0.0190 | * |
| Fen 2.6 µM, 0Gy | Fen 2.6 µM, 10Gy | 0.1132 | ns |

**Table 2** Statistical analysis of MFI data using paired Students t-test

| **Treatment A** | **Treatment B** | **p-Value** | **Significance** |
| --- | --- | --- | --- |
| **PAN-ROS NAC** | | | |
| DMSO, 0Gy | DMSO, 2Gy | 0.0343 | * |
| DMSO, 0Gy | DMSO, 5Gy | 0.0300 | * |
| DMSO, 2Gy | Fen 0.5 µM, 2Gy | 0.0042 | ** |
| DMSO, 2Gy | Fen 1.4 µM, 2Gy | 0.0125 | * |
| DMSO, 5Gy | Fen 0.5 µM, 5Gy | 0.0114 | * |
| DMSO, 5Gy | Fen 1.4 µM, 5Gy | 0.0045 | ** |
| Fen 0.5 µM, 0Gy | Fen 0.5 µM, 2Gy | 0.0053 | ** |
| Fen 0.5 µM, 0Gy | Fen 0.5 µM, 5Gy | 0.0062 | ** |
| Fen 1.4 µM, 0Gy | Fen 1.4 µM, 2Gy | 0.0099 | ** |
| Fen 1.4 µM, 0Gy | Fen 1.4 µM, 5Gy | 0.0119 | * |
| DMSO, 0Gy | NAC, DMSO, 0Gy | 0.0043 | ** |
| Fen 0.5 µM, 0Gy | NAC, Fen 0.5 µM | 0.0403 | * |
| Fen 1.4 µM, 0Gy | NAC, Fen 1.4 µM | 0.0123 | * |
| DMSO, 2Gy | NAC, DMSO, 2Gy | 0.0018 | ** |
| Fen 0.5 µM, 2Gy | NAC Fen 0.5 µM, 2Gy | 0.0405 | * |
| Fen 1.4 µM, 2Gy | NAC Fen 1.4 µM 2Gy | 0.0123 | * |
| DMSO, 5Gy | NAC, DMSO, 5Gy | 0.0148 | * |
| Fen 0.5 µM, 5Gy | NAC Fen 0.5 µM 5Gy | 0.0308 | * |
| Fen 1.4 µM, 5Gy | NAC Fen 1.4 µM, 5Gy | 0.0182 | * |

**Table 3** Statistical analysis of MFI data using paired Students t-test

| **Treatment A** | **Treatment B** | **p-Value** | **Significance** |
| --- | --- | --- | --- |
| **MitoSox** | | | |
| DMSO, 0Gy | DMSO, 2Gy | 0.5531 | ns |
| DMSO, 0Gy | DMSO, 5Gy | 0.0249 | * |
| Fen 0.5 µM, 0Gy | Fen 0.5 µM, 2Gy | 0.0523 | ns |
| Fen 0.5 µM, 0Gy | Fen 0.5 µM, 5Gy | 0.0289 | * |
| Fen 1.4 µM, 0Gy | Fen 1.4 µM, 2Gy | 0.1624 | ns |
| Fen 1.4 µM 0Gy | Fen 1.4 µM, 5Gy | 0.0036 | ** |

**Table 4** Statistical analysis of cell viability using paired Students t-test

| **Treatment A** | **Treatment B** | **p-Value** | **Significance** |
| --- | --- | --- | --- |
| **Different Inhibitors** | | | |
| DMSO, 0Gy | 1.4 µM Fen, 0Gy | 0.5022 | ns |
| DMSO, 0Gy | DMSO, 2Gy | 0.1198 | ns |
| DMSO, 0Gy | Fen 1.4 µM, 2Gy | 0.0459 | * |
| Fen 1.4 µM, 0Gy | VitC, Fen 1.4 µM, 0Gy | 0.3080 | ns |
| Fen 1.4 µM, 0Gy | MT, Fen 1.4 µM, 0Gy | 0.9648 | ns |
| DMSO, 2Gy | VitC, DMSO, 2Gy | 0,1674 | ns |
| DMSO, 2Gy | MT, DMSO, 2Gy | 0,3227 | ns |
| Fen 1.4 µM, 2Gy | VitC, Fen 1.4 µM 2Gy | 0,2351 | ns |
| Fen 1.4 µM, 2Gy | MT, Fem 1.4 µM, 2Gy | 0,8130 | ns |
| VitC, DMSO, 0Gy | VitC, Fen 1.4 µM, 0Gy | 0,2199 | ns |
| VitC, DMSO, 0Gy | VitC, DMSO, 2Gy | 0,9317 | ns |
| VitC, DMSO, 0Gy | VitC, Fen 1.4 µM, 2Gy | 0,6376 | ns |
| MT, DMSO, 0Gy | MT, Fen 1.4 µM, 0Gy | 0,1270 | ns |
| MT, DMSO, 0Gy | MT, DMSO, 2Gy | 0,6383 | ns |
| MT, DMSO, 0Gy | MT, Fen 1.4 µM, 2Gy | 0,1036 | ns |

**Table 5** Statistical analysis of cell viability using paired Students t-test, AO 530/30

| **Treatment A** | **Treatment B** | **p-Value** | **Significance** |
| --- | --- | --- | --- |
| **Different Inhibitors** | | | |
| DMSO, 0Gy | Dyn, 0Gy | 0.1034 | ns |
| Fen 1.4 µM, 0Gy | Dyn, Fen 1.4 µM, 0 Gy | 0.2165 | ns |
| Fen 3 µM, 0Gy | Dyn, Fen 3 µM,0 Gy | 0.0228 | * |
| DMSO, 2Gy | Dyn, 2Gy | 0.1077 | ns |
| Fen 1.4 µM, 2Gy | Dyn, Fen 1.4 µM, 2 Gy | 0.1500 | ns |
| Fen 3 µM, 2Gy | Dyn, Fen 3 µM, 2 Gy | 0.0073 | ** |
| DMSO, 5Gy | Dyn, 5Gy | 0.0623 | ns |
| Fen 1.4 µM, 5Gy | Dyn, Fen 1.4 µM, 5Gy | 0.0119 | * |
| Fen 3 µM, 5Gy | Dyn, Fen 3 µM, 5 Gy | 0.0274 | * |
| DMSO, 0Gy | VitC, 0Gy | 0.7328 | ns |
| Fen 1.4 µM, 0Gy | VitC, Fen 1.4 µM, 0 Gy | 0.0697 | ns |
| Fen 3 µM, 0Gy | VitC, Fen 3 µM,0 Gy | 0.0165 | * |
| DMSO, 2Gy | VitC, 2Gy | 0.1942 | ns |
| Fen 1.4 µM, 2Gy | VitC, Fen 1.4 µM, 2 Gy | 0.0430 | * |
| Fen 3 µM, 2Gy | VitC, Fen 3 µM, 2 Gy | 0.0107 | * |
| DMSO, 5Gy | VitC, 5Gy | 0.7704 | ns |
| Fen 1.4 µM, 5Gy | VitC, Fen 1.4 µM, 5Gy | 0.0321 | * |
| Fen 3 µM, 5Gy | VitC, Fen 3 µM, 5 Gy | 0.0188 | * |
| DMSO, 0Gy | Z-vad, 0Gy | 0.9831 | ns |
| Fen 1.4 µM, 0Gy | Z-vad, Fen 1.4 µM, 0 Gy | 0.4557 | ns |
| Fen 3 µM, 0Gy | Z-vad, Fen 3 µM,0 Gy | 0.4776 | ns |
| DMSO, 2Gy | Z-vad, 2Gy | 0.3716 | ns |
| Fen 1.4 µM, 2Gy | Z-vad, Fen 1.4 µM, 2 Gy | 0.0059 | ** |
| Fen 3 µM, 2Gy | Z-vad, Fen 3 µM, 2 Gy | 0.0796 | ns |
| DMSO, 5Gy | Z-vad, 5Gy | 0.8733 | ns |
| Fen 1.4 µM, 5Gy | Z-vad, Fen 1.4 µM, 5Gy | 0.2504 | ns |
| Fen 3 µM, 5Gy | Z-vad, Fen 3 µM, 5 Gy | 0.2121 | ns |

**Table 6** Statistical analysis of cell viability using paired Students t-test, AO 670/30

| **Treatment A** | **Treatment B** | **p-Value** | **Significance** |
| --- | --- | --- | --- |
| **Different Inhibitors** |  |  |  |
| DMSO, 0Gy | Dyn, 0Gy | 0.0029 | ** |
| Fen 1.4 µM, 0Gy | Dyn, Fen 1.4 µM, 0 Gy | 0.3724 | ns |
| Fen 3 µM, 0Gy | Dyn, Fen 3 µM,0 Gy | 0.0007 | *** |
| DMSO, 2Gy | Dyn, 2Gy | 0.0002 | *** |
| Fen 1.4 µM, 2Gy | Dyn, Fen 1.4 µM, 2 Gy | 0.0027 | ** |
| Fen 3 µM, 2Gy | Dyn, Fen 3 µM, 2 Gy | 0.0002 | *** |
| DMSO, 5Gy | Dyn, 5Gy | 0.0062 | ** |
| Fen 1.4 µM, 5Gy | Dyn, Fen 1.4 µM, 5Gy | 0.0092 | ** |
| Fen 3 µM, 5Gy | Dyn, Fen 3 µM, 5 Gy | 0.0023 | ** |
| DMSO, 0Gy | VitC, 0Gy | 0.0634 | ns |
| Fen 1.4 µM, 0Gy | VitC, Fen 1.4 µM, 0 Gy | 0.0614 | ns |
| Fen 3 µM, 0Gy | VitC, Fen 3 µM,0 Gy | 0.0009 | *** |
| DMSO, 2Gy | VitC, 2Gy | 0.7648 | ns |
| Fen 1.4 µM, 2Gy | VitC, Fen 1.4 µM, 2 Gy | 0.0815 | ns |
| Fen 3 µM, 2Gy | VitC, Fen 3 µM, 2 Gy | 0.0003 | *** |
| DMSO, 5Gy | VitC, 5Gy | 0.3619 | ns |
| Fen 1.4 µM, 5Gy | VitC, Fen 1.4 µM, 5Gy | 0.1516 | ns |
| Fen 3 µM, 5Gy | VitC, Fen 3 µM, 5 Gy | 0.0004 | *** |
| DMSO, 0Gy | Z-vad, 0Gy | 0.9825 | ns |
| Fen 1.4 µM, 0Gy | Z-vad, Fen 1.4 µM, 0 Gy | 0.8027 | ns |
| Fen 3 µM, 0Gy | Z-vad, Fen 3 µM,0 Gy | 0.4799 | ns |
| DMSO, 2Gy | Z-vad, 2Gy | 0.9556 | ns |
| Fen 1.4 µM, 2Gy | Z-vad, Fen 1.4 µM, 2 Gy | 0.3011 | ns |
| Fen 3 µM, 2Gy | Z-vad, Fen 3 µM, 2 Gy | 0.2462 | ns |
| DMSO, 5Gy | Z-vad, 5Gy | 0.2761 | ns |
| Fen 1.4 µM, 5Gy | Z-vad, Fen 1.4 µM, 5Gy | 0.7913 | ns |
| Fen 3 µM, 5Gy | Z-vad, Fen 3 µM, 5 Gy | 0.0214 | * |

**Drugs and Stains**

**Table 7** Drugs used in the experiments

|  | **Product information** |
| --- | --- |
| Dynasore hydrate (dynasore) | D7693, Sigma |
| L-ascorbic acid (Vitamin C) | A4403, Sigma |
| MitoTempo (MT) | SML0737, Sigma |
| Retinoic acid p-hydroxyanilide (fenretinide, Fen) | H7779, Sigma |
| Z-vad-FMK | A1902, APExBIO |
| N-acetylcysteine | A7250, Sigma |

**Table 8** Fluorescent stains for flow cytometry and fluorescence microscopy

|  | **Product information** |
| --- | --- |
| Acridine Orange (hemi zinc chloride salt) | 158550, Sigma |
| CellRox Deep Red Reagent | C10422, Thermo Fisher |
| Lucifer Yellow | L453, Thermo Fisher |
| MitoSox Red | M36008, Thermo Fisher |
| Propidium Iodide | 4170, Sigma |
