# Supplementary figures and images for: Fenretinide Acts as Potent Radiosensitizer for Treatment of Rhabdomyosarcoma Cells
Source: Front Oncol. 2021 Jun 15;11:664462. doi: 10.3389/fonc.2021.664462 (PMC8239363; doi:10.3389/fonc.2021.664462)

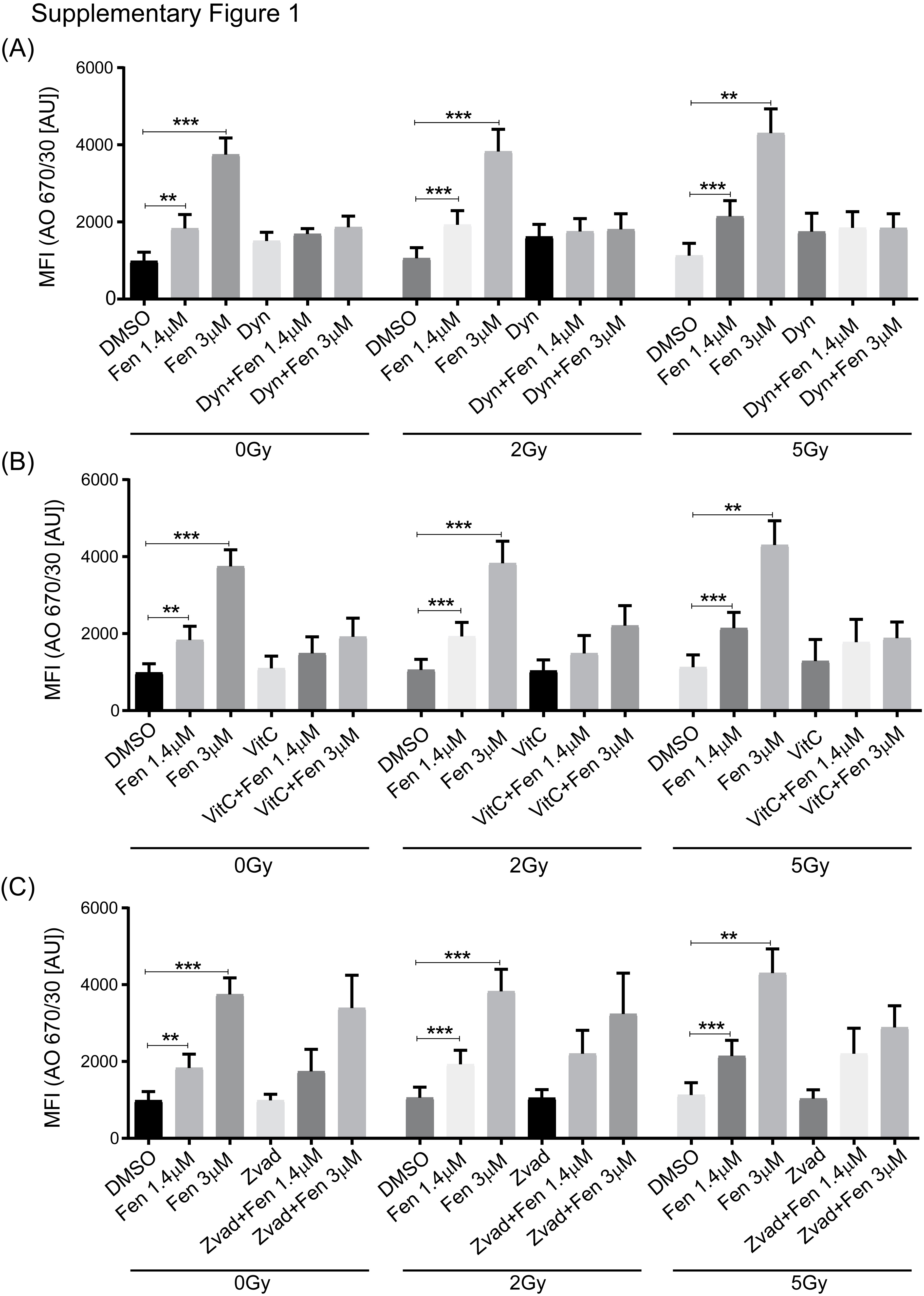

Supplement: Supplementary file 2 [file Image_1.jpeg]
